# Bicellular Tight Junctions and Wound Healing

**DOI:** 10.3390/ijms19123862

**Published:** 2018-12-04

**Authors:** Junhe Shi, May Barakat, Dandan Chen, Lin Chen

**Affiliations:** 1 Center for Wound Healing and Tissue Regeneration, College of Dentistry, University of Illinois at Chicago, 801 S. Paulina Street, Chicago, IL 60612, USA; jshi36@uic.edu (J.S.); mbarak5@uic.edu (M.B.); 2 Colgate-Palmolive Company, Piscataway, NJ 08855, USA; dandan_chen@colpal.com

**Keywords:** tight junctions, occludin, claudin, junctional adhesion molecule, zonula occludens, wound healing

## Abstract

Bicellular tight junctions (TJs) are intercellular junctions comprised of a variety of transmembrane proteins including occludin, claudins, and junctional adhesion molecules (JAMs) as well as intracellular scaffold proteins such as zonula occludens (ZOs). TJs are functional, intercellular structures that form a barrier between adjacent cells, which constantly seals and unseals to control the paracellular passage of molecules. They are primarily present in the epithelial and endothelial cells of all tissues and organs. In addition to their well-recognized roles in maintaining cell polarity and barrier functions, TJs are important regulators of signal transduction, which modulates cell proliferation, migration, and differentiation, as well as some components of the immune response and homeostasis. A vast breadth of research data is available on TJs, but little has been done to decipher their specific roles in wound healing, despite their primary distribution in epithelial and endothelial cells, which are essential contributors to the wound healing process. Some data exists to indicate that a better understanding of the functions and significance of TJs in healing wounds may prove crucial for future improvements in wound healing research and therapy. Specifically, recent studies demonstrate that occludin and claudin-1, which are two TJ component proteins, are present in migrating epithelial cells at the wound edge but are absent in chronic wounds. This indicates that functional TJs may be critical for effective wound healing. A tremendous amount of work is needed to investigate their roles in barrier function, re-epithelialization, angiogenesis, scar formation, and in the interactions between epithelial cells, endothelial cells, and immune cells both in the acute wound healing process and in non-healing wounds. A more thorough understanding of TJs in wound healing may shed new light on potential research targets and reveal novel strategies to enhance tissue regeneration and improve wound repair.

## 1. Introduction

**Figure 1:**
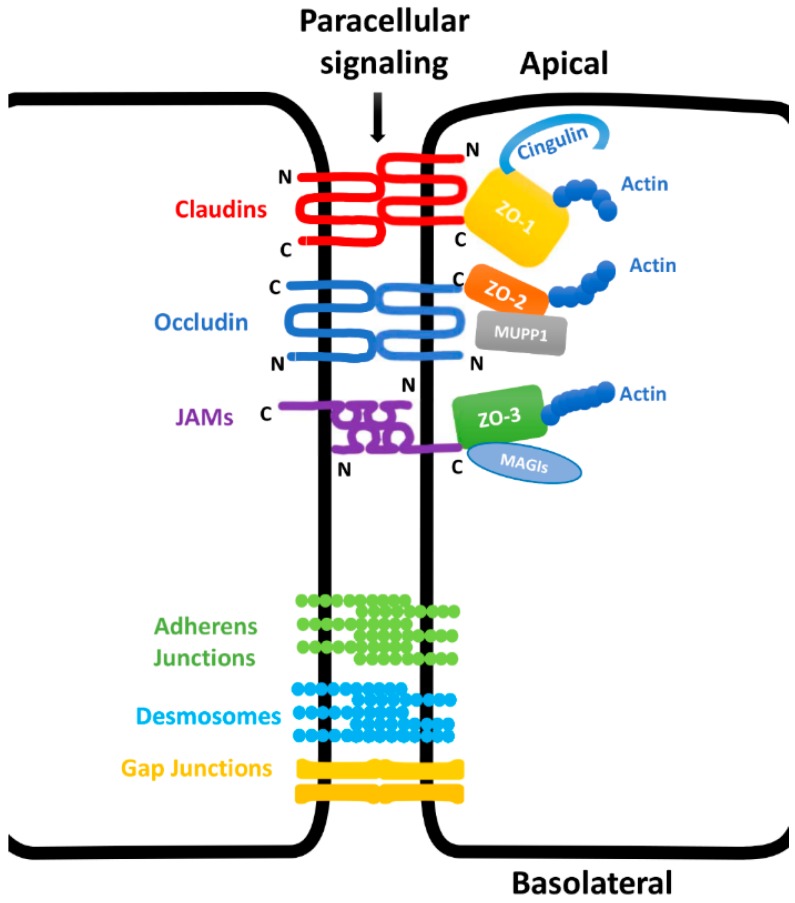
Schematic structures of major bicellular tight junction proteins. The tight junction (TJ) is part of cell-cell junction complex. Major bicellular TJ proteins include three transmembrane protein families: occludin, claudins, and junctional adhesion molecules (JAMs) and a few families of peripheral intracellular membrane proteins such as zonula occludens (ZOs), which connect the transmembrane TJ molecules to the actin filament cytoskeleton.

Tight junctions are one of several types of cell-cell junctions, including gap junctions, desmosomes, and adherens junctions. TJs comprise more than 40 molecules derived from three integral transmembrane protein families: occludin, claudins, and junctional adhesion molecules (JAMs), as well as a few families of peripheral intracellular membrane proteins, which form bridges between the transmembrane tight junction (TJ) molecules and the actin filament cytoskeleton. These proteins include the scaffold post-synaptic density 95/Drosophila discs large/zona-occludens (PDZ) -domain proteins such as zonula occludens proteins (ZOs) 1, 2, and 3; multiple-PDZ domain protein 1 (MUPP1); membrane-associated guanylate kinase isoforms (MAGIs) 1, 2, and 3; cell polarity proteins, such as atypical protein kinase C (PKC); isotype-specific-interacting protein/protease-activated receptor-3 (ASIP/PAR-3) and PAR-6; and the non-PDZ-expressing proteins, such as cingulin, symplekin, atypical protein kinase C (aPKC), protein phosphatase 2 (PP2A), Ras-related protein Rab-3B (Rab3b) and Rab13 [1,2,3,4,5,6] (Figure 1).

TJ molecules extend from adjacent cells to form paired strands that seal the paracellular pathway. These connections are the gates for the TJ’s essential barrier function and control the passage of ions and molecules via charge and size selectivity. Molecules that meet specific criteria are allowed to pass between cells by entering on the apical side, passing through the TJ protein strands, and exiting on the basolateral side (Figure 1) [6,7]. These protein components also allow TJs to maintain cell polarity and prevent the diffusion and interaction of molecules in the apical membrane with those in the lateral membrane. This is a key role known as the fence function [5]. In addition to their barrier and fence functions, TJs play significant roles in signal transduction and in the function of immune cells [8]. Some proteins within TJs can function as receptors for viral or pathogenic bacterial antigens like *Clostridium perfringens* enterotoxin, whose carboxyl terminal half-fragments can selectively bind to and remove claudin-4 from TJ strands, disrupting the barrier function of epithelial cells [9].

Given their broad array of functions, TJs are present throughout the body, especially in endothelial and epithelial cells, which are critically involved in wound healing. The wound healing process has four overlapping phases: hemostasis, inflammation, proliferation, and tissue remodeling. Many cell types, including keratinocytes, epithelial cells, endothelial cells, fibroblasts, adipocytes; resident leukocytes, such as dendritic epidermal γδ T cells (DETCs), mast cells, and Langerhans cells; and infiltrating leukocytes, such as neutrophils, macrophages, and lymphocytes, including CD4/CD8 T cells, B cells, regulatory T cells, and natural killer T (NKT) cells; play pivotal roles in the wound healing process [10,11]. The migration, proliferation, and differentiation of these cells are tightly regulated by numerous factors, including inflammatory cytokines and chemokines, growth factors, proteinases, and hormones [10,11,12]. While it is known that TJs are present in several of these crucial cell populations and can regulate many of the cellular functions and interactions involved in wound healing, little work has been done to elucidate their specific contributions. In this paper, we will discuss several extensively studied bicellular TJ proteins, including occludin, claudins, JAMs, and ZOs, and present the limited knowledge currently available regarding their roles in wound healing to highlight the importance of further research in this area. It is important to note that, while tri-cellular TJs, including tricellulin and the angulin family of proteins (angulin-1, 2, and 3), are also an important barrier structure [13], their contribution to wound healing is totally unknown. Therefore, they will not be addressed in this review.

## 2. Occludin

Occludin is a transmembrane protein with a molecular mass of 65 kDa. Occludin, along with MarvelD2 (tricellulin) and MarvelD3, belongs to the TJ-associated marvel protein family [14]. Occludin contains four transmembrane domains, two extracellular loops, and three cytoplasmic domains [15]. The C-terminal domain directly binds to ZOs, which subsequently interact with the actin cytoskeleton (Figure 1) [16]. Overexpression of occludin decreases permeability in Madin-Darby canine kidney (MDCK) epithelial cells, as indicated by an increase in transepithelial electronic resistance (TEER). This is a measurement of the electrical resistance across a monolayer of cells, which serves as an indicator of monolayer integrity and permeability [17].

In contrast to the overexpression phenotype, deletion of the occludin C-terminal domain leads to impaired fence function in epithelial cells. While occludin knockout mice are born with no immediately apparent abnormalities, they show significant postnatal growth retardation. The morphology of TJs, including component proteins claudin-4, ZO-1, and ZO-2, does not appear to be altered in occludin knockout mice, and no barrier function abnormalities are observed in the intestinal epithelium. However, various histological abnormalities are observed in other tissues, including chronic inflammation, hyperplasia of the gastric epithelium, atrophic testes, and the loss of cytoplasmic granules in salivary gland duct cells, indicating that occludin has a complex variety of functions [18]. Occludin is also associated with several cellular proteins and can be modulated by important signaling pathways. Occludin serves as an initial receptor for GTPase signaling, and increased phosphorylation of occludin proteins decreases TJ permeability [19]. Furthermore, Itch, which is an E3 ubiquitin-protein ligase, can bind to the N-terminus of occludin. This indicates that occludin may be regulated by ubiquitination [20]. The Ras/rapidly accelerated fibrosarcoma/mitogen-activated protein kinase/extracellular signal-regulated kinase (Ras-Raf-MEK-ERK) signaling pathway is also important in occludin function. Raf-1 is a downstream mediator in the Ras signaling pathway. Overexpression of Raf-1 in epithelial cells reduces the expression of occludin, disrupts junctional proteins, changes the localization of the actin cytoskeleton, and induces cell stratification. Taken together, these studies suggest that occludin is associated with multiple cellular proteins and can be modulated by a variety of signaling pathways [19]. Because these pathways can affect apoptosis, signal transduction, cell growth and proliferation, and other critical factors in healing, these results further suggest that occludin may play a significant role in the wound healing process.

## 3. Claudins

Claudins belong to a superfamily currently comprised of 27 small transmembrane proteins in humans/mammals [21,22,23] and 67 in fish [24]. Claudins have a relative molecular mass of 20 to 27 kDa. Each claudin protein has two extracellular loops, one intracellular loop, and N-terminal and C-terminal cytoplasmic domains [25,26]. Claudins also have a PDZ-binding motif at their C-terminus, which can bind to ZOs or MUPP-1 (Figure 1) [27,28,29]. Claudins regulate conductance through the paracellular pathway by size and charge selection. Individual claudin proteins are generally classified as either barrier-forming or pore-forming (channel-forming) claudins based on whether their expression increases or decreases permeability, which is measured by TEER. For instance, over-expression of claudin-1 and 4 in MDCK epithelial cells significantly increases TEER, which indicates reduced permeability [30,31]. Reduction of claudin-1 expression in human skin keratinocytes results in decreased TEER and indicates increased permeability [32]. SiRNA knockdown of Claudin-1 also reduces TEER and increases paracellular tracer flux for sodium fluorescein, which indicates disruption of the barrier function [33]. Therefore, claudin-1 and 4 are classified as barrier claudins. Other barrier claudins include claudins 5, 6, 8, 9, 11, 15, and 19 [22,34,35]. In contrast, over-expression of claudin-2, which is a pore-forming claudin, results in a markedly decreased TEER in MDCK cells [36]. Other pore-forming claudins include claudins 10, 15, and 17. Claudins 2, 10b, and 15 are cation pores, while claudins 10a and 17 are anion pores [34]. Furthermore, studies demonstrate that variations in claudin expression can alter the assembly and function of TJs in the epithelium. Epidermal overexpression of claudin-6 in transgenic mice significantly increases trans-epidermal water loss, which results in death within 48 h of birth. Overexpression of claudin-6 also downregulates expression of other claudins, which suggests a negative feedback loop between existing claudins and subsequent claudin expression. In these claudin-6 overexpressing mice, the epidermis also demonstrates abnormal expression of differentiation markers, such as keratin 1, filaggrin, loricrin, and involucrin [37]. These studies begin to detail the large number of claudins and their variable and complex functions. However, the mechanisms underlying both their involvement in the occlusion of the paracellular cleft and the distinction between barrier-forming and pore-forming claudins are beyond the scope of this review. Readers areadvised to refer to a few outstanding reviews cited for further information [22,34,35].

In addition to their inherent effects on permeability, claudins can also affect barrier activity after phosphorylation. The C-terminal region of the claudin cytoplasmic domain contains several phosphorylation sites regulated by protein kinases such as mitogen-activated protein kinases (MAPKs) and others that rely on cyclic adenosine monophosphate [38]. Phosphorylation of some claudin proteins, such as claudin-1, in MDCK cells causes severe damage to TJs, resulting in increased cellular permeability [31]. The activation of PKC also affects the expression, localization, and phosphorylation of claudins and, consequently, regulates the epithelial barrier function [26,39]. These studies on claudin expression and involvement in signaling pathways indicate that claudins are important for normal epithelial function, further suggesting that they may play significant roles in proper wound healing due to the involvement of the epithelium itself and the associated signaling pathways in injury and the healing process.

Beyond their functions and expression in epithelial tissues, claudins have also been identified in tissue-specific distributions in the endothelium. For example, human and mouse brain endothelial cells express claudins 3, 5, and 12, while kidney endothelial cells express claudins 5 and 15 [26]. In mouse placenta, the expression levels of claudins 1, 2, 4, and 5 are increased when compared to other claudin family members [40]. Claudin-5 is also present in the vascular endothelia of the skin, brain, and lung in mice [41,42]. Because the epithelium and endothelium are directly involved in dermal injury and repair, the effects of claudins on epithelial and endothelial function may impact the wound healing process.

## 4. Junctional Adhesion Molecules

JAMs belong to the immunoglobulin superfamily and were the first TJ molecules identified in both endothelial and epithelial cells [43]. JAMs have three unique structural domains, including an extracellular segment with two variable type immunoglobulin-like domains and a single transmembrane section with a C-terminal intracellular domain (Figure 1) [43]. All three JAMs contain tyrosine and serine/threonine on their cytoplasmic ends. Despite the early discovery of JAMs, the effects of their involvement in signal transduction on the regulation of TJ functions are not well studied compared to other TJ proteins.

The JAM family is comprised of two groups [44]. The first group includes JAM-A/F11r/CD321, JAM-B/CD322, and JAM-C/CD323 and features a class II PDZ domain-binding motif at the C-terminus, which binds to ZO-1 and PAR-3. The other group has a class I PDZ domain-binding motif at the C-terminus [27,45,46] and includes the coxsackie and adenovirus receptor (CAR), endothelial cell-selective adhesion molecule (ESAM), and JAM-D. JAMs are often associated with the recruitment of other TJ molecules, such as occludin, and intracellular binding partner PDZ domain proteins, such as ZO-1, AF-6, CASK, PAR-3, and MUPP-1 [43,44,46,47]. Together, these partners may play an important role in TJ assembly, reorganization of the actin cytoskeleton, and regulation of cell polarity [48]. JAMs also play an important role in the barrier function of TJs and the development of apico-basal cell polarity in epithelial cells [43]. For instance, one study demonstrates that antibodies to JAM-A increase monolayer permeability in a human retinal pigment epithelial cell line [49]. Additionally, the inhibition of JAM-A reduces fibroblast growth factor (FGF) 2-induced proliferation and migration of endothelial cells [50]. FGF-2 also fails to induce micro-vessel sprouting in JAM-A null mice [51], which indicates that endothelial cell JAM expression may be important for proper angiogenesis. Furthermore, mouse dermal blood vessels exhibit significantly reduced leukocyte infiltration in contact-dermatitis-induced skin inflammation when treated with neutralizing antibodies against JAM-B and JAM-C. This indicates that JAMs are involved in the inflammation and regulate leukocyte infiltration [52]. Taken together, these results demonstrate that JAMs are involved in both angiogenesis and inflammation, which are two major components of the wound healing process.

## 5. Zonula Occludens

Zonula occludens proteins are peripherally associated cytoplasmic membrane proteins that belong to the membrane associated guanylate kinase homologue (MAGUK) protein family. Three distinct MAGUKs have been identified: ZO-1, ZO-2, and ZO-3, with molecular weights of 220, 160, and 130 kDa, respectively. ZO-1, which was discovered in 1986, was the first identified member of the TJ family [53]. The main features of the ZO protein family are their three structurally conserved PDZ domains, one Src homology 3 domain, and one guanylate kinase domain. ZOs anchor transmembrane proteins, such as claudins, occludin, and JAMs, to the actin cytoskeleton (Figure 1) [54,55] and are expressed in key cell types involved in the wound healing process. ZO-1 and ZO-2 are expressed in both the epithelium and the endothelium, while ZO-3 is only expressed in the epithelium [56].

The functions of ZOs have been examined using deletion strategies. Absence of ZO-1 in mouse mammary gland epithelial cells leads to delayed recruitment of claudins and occludin to TJs and postponed barrier establishment [57]. In MDCK cells, siRNA knockdown of ZO-2 results in increased paracellular permeability, decreased TEER and fence function of TJs, and defective recruitment of ZO-1, occludin, and E-cadherin to recently formed junctions [58]. ZO-1 and ZO-2 knockouts have embryonic lethal qualities [59,60]. ZO-1 knockouts are associated with a defective yolk sac angiogenesis and apoptosis of embryonic cells [59]. Embryos of ZO-2 knockout mice exhibit reduced proliferation, increased apoptosis, and increased paracellular permeability, which indicates a disrupted barrier function [60]. Another study demonstrated that ZO-2 knockout results in a damaged blood-testis barrier [61]. Embryos of ZO-3 knockout mice demonstrate no observed alterations in the phenotype, which indicates that ZO-3 is potentially a dispensable component of TJs [60].

Several research papers have also shown that ZOs, unlike other TJ component proteins, can shuttle between the cytoplasm and the nucleus, especially in response to injury or stressors. For example, ZO-2 accumulates in the nucleus in MDCK epithelial cells following heat shock or chemical insult [62]. Mechanical injury, such as scratch infliction, also induces ZO-2 nuclear localization due to damage to cell-cell contacts [63], highlighting the possibility that ZOs may play significant roles in an injured or damaged environment like that of a healing wound. Furthermore, substantial work suggests that ZO-2 and other ZOs interact with a variety of nuclear proteins, including transcription factors, to modulate the growth and proliferation of epithelial and endothelial cells, which are both essential to the wound healing process. For more detailed information, please refer to the review by Bauer et al [54].

ZOs can undergo post-translational modification with phosphate, and both PKA and PKC are responsible for increased phosphorylation of ZO-2 [64]. Hyper-phosphorylation of ZO-2 correlates with increased TEER, which indicates decreased permeability and can be corrected by treatment with a PKC inhibitor [64]. Similarly, ZO-1 phosphorylation is reduced in response to PKC inhibitors, which induce the assembly of TJs by increasing calcium concentrations in the cell culture media [65]. ZO proteins can also be tyrosine phosphorylated in conditions that favor the assembly of TJs or that weaken TJ barrier function [26]. These studies demonstrate the important role of ZOs in cell signaling post-injury in the recruitment and anchoring of other transmembrane TJ proteins and in the overall integrity of the TJ complex. Collectively, these investigations into the functions of ZOs reveal multiple possibilities that remain to be deciphered regarding their involvement in wound healing.

## 6. Tight Junctions in the Skin

The skin is often considered the largest organ in the human body. Given the fact that the skin is the first line of defense against the hostile external environment, the integrity of TJs in the epidermis is critical due to their fence and barrier functions and their roles in homeostasis and the immune response. The epidermis in humans and in mice expresses a variety of TJ molecules, the locations of which are summarized in Table 1. For example, human skin expresses claudins 1, 3, 4, 5, 7, 8, 10, 11, 12, 16, and 17, occludin, JAM-A, JAM-B, ZO-1, cingulin, MUPP-1, and symplekin [66]. Of those, claudin-1, claudin-7, JAM-A, and MUPP1 are found in all layers of the epidermis, while occludin and cingulin are limited to the stratum granulosum [66]. JAM-A is expressed in all layers of the human epidermis except the stratum corneum [2]. In the adult mouse epidermis, occludin primarily localizes to the most superficial zone of the granular layer, while ZO-1 and ZO-2 are present from the spinous to the granular layers [67]. Some TJ proteins, such as ZO-1, claudin-1, and claudin-4, also localize to the hair follicles [67,68].

TJs are critical for the skin barrier function, and changes in their expression have been identified in several abnormal skin conditions, which emphasizes the possibility that they may be important for normal skin wound healing. Claudin-1 knockout mice die shortly after birth due to skin-barrier defects leading to severe water loss [69]. Knockdown of claudins 1 and 4, occludin, and ZO-1 in skin keratinocytes increases paracellular permeability for ions and larger molecules. In addition, knockdown of claudin-1 also reduces the water barrier function in the stratum corneum [70]. In atopic dermatitis, ZO-1 and claudin-4 are significantly reduced in non-lesional skin. ZO-1 and claudin-1 are significantly reduced in lesional skin, which suggests that the TJ barrier function is impaired in this skin condition [71,72]. Another study in a mouse model of atopic dermatitis showed that the changes in skin epithelial barrier function correlated with a decreased expression of claudin-1, as indicated by changes in TEER, paracellular flux, and the morphology of the stratum corneum [73]. In early-phase psoriasis, occludin, ZO-1, and claudin-4 were present in more epidermal layers than in normal skin, and the expression of claudins 1 & 7 was reduced in the basal and the uppermost layers [74]. Claudin expression was further decreased in plaque-type psoriasis and these changes may contribute to the impaired barrier function of psoriatic skin [74].

In addition to their barrier functions for water, ions, and large and small molecules, TJs are also involved in cell proliferation and differentiation. In human skin keratinocytes, siRNA knockdown of Claudin-1 disrupts TJ function and results in decreased TEER, increased permeability to sodium fluorescein, and increased proliferation [33]. In an atopic dermatitis mouse model, the absence of claudin-1 in the lower epidermal layers correlates with significantly increased proliferation of epithelial cells, as well as changes in the expression of differentiation markers keratin-10 and keratin-14 [75]. Furthermore, knockdown of occludin in human skin keratinocytes results in decreased epithelial cell-cell adhesion, reduced susceptibility to apoptosis, and altered expression of differentiation markers [76]. For more information, please see references [3,77]. Taken together, these results highlight the involvement of TJs in a variety of skin disorders and the downstream consequences of abnormal TJ expression on normal skin function, further underscoring the potential roles TJ proteins may play in skin injury and repair.

## 7. Tight Junctions in the Oral Mucosa

Like the skin, the oral mucosa endures regular exposure to the hostile external environment. Based on differences in histology, localization, and specific function, the oral mucosa is categorized into three types: lining mucosa, masticatory mucosa, and specialized mucosa. Lining mucosa is a non-keratinized, stratified, squamous epithelium and is widely present on the labial, buccal, and alveolar mucosa and throughout the mouth. Masticatory mucosa is a keratinized, stratified, squamous epithelium and is present on the gingiva, the dorsum of the tongue, and the hard palate. Like the skin epidermis, it contains four layers of stratified squamous cells. Specialized mucosa is localized to the taste buds and papillae of the tongue and functions in taste and sensory perception [91,92].

All three of these mucosal types exist within the oral cavity, which has a complex microbiome. Oral epithelium faces the constant challenge of protecting against microorganisms and other insults including burns, cold temperatures, and mechanical injuries, much like the skin serves as the first line of protection in the immune system. Since the oral mucosa serves such a similar purpose to the skin, it is expected that the barrier functions of TJ proteins also play a crucial role in its function. However, there is currently very limited information available regarding the locations (Table 2) and functions of TJs in in vivo oral tissues. Minimally inflamed gingival tissue uniformly expresses ZO-1, ZO-2, occludin, JAM-A, claudin-4, and claudin-15, while the pocket epithelium of periodontal lesions shows only scattered staining for these TJ proteins [93], suggesting that their expression decreases in damaged or unhealthy tissue. In human gingival keratinocyte cell line Gie-3B11, treatment with micronutrients such as Quercetin, Acetyl-11-keto-β-boswellic acid (AKBA), and retinoic acid increases expression of claudins 2, 4, and 5 and leads to increased TEER and decreased permeability to ^14^C-d-mannitol [94]. These results indicate that the induced changes in claudin expression alter barrier function in these cells.

However, while the function of the oral mucosa as a protective barrier is similar to that of the skin, it is known that there are key differences between them, especially during wound healing and tissue repair [95,96,97]. Normal skin and oral keratinocytes have different barrier functions under the same conditions, as shown by an in vitro study that demonstrated that gingival keratinocytes have higher TEER than HACAT cells that are part of an immortalized human skin keratinocyte cell line [98]. Together, these results highlight the differences between the skin and the oral mucosa and further emphasize the importance of deciphering the specific functions of TJ proteins. Because the oral mucosa is known to exhibit superior wound healing compared to the skin, a deeper understanding of the roles TJs protein play in the function of the oral mucosa may also shed light on their involvement in the wound healing process itself.

## 8. Tight Junctions in Immune Cells

In addition to epithelial and endothelial cells, immune cells also express functional TJ proteins. Several human non-neutrophil leukocyte subsets, including B-lymphocytes, CD4+ and CD8+ lymphocytes, and monocytes, express claudin-1 and claudin-5 [100]. In vitro activation of these immune cells increases the expression levels of claudin-1 but not claudin-5 [100]. DETCs, which recognize signals from stressed or damaged keratinocytes, play a pivotal role in the skin’s innate immune response. They secrete insulin-like growth factor 1 (IGF-1) and keratinocyte growth factors (KGFs) after activation and are critical for skin wound repair [101]. One study shows that migration of activated DETCs into draining lymph nodes is dependent on occludin expression, and DETCs in occludin-deficient mice have impaired motility and display morphological changes [102]. These data suggest that occludin expression in stress-activated DETCs is important for proper migration and may be critical for key interactions between DETCs and other cells that modulate the immune response. One key immune process during inflammation is the migration of neutrophils to an affected area through a series of receptor-mediated steps. On their surface, neutrophils express a JAM-like protein (JAML) which interacts with the CAR on epithelial cells to mediate neutrophil migration across the epithelial TJs [103]. JAM extracellular domains are capable of both homophilic and heterophilic binding with β1 and β2 integrins, including αLβ2 (LFA-1), α4β1 (VLA-4), and αMβ2 (Mac-1), which are present on circulating leukocytes and platelets [44,47]. These are cell types that are essential for mounting a normal immune response. JAMs are also present on endothelial cells and epithelial cells and may mediate cell–cell adhesion between many of the different cell types involved in inflammation. For example, JAM-A-deficient mice exhibit reduced neutrophil diapedesis in peritonitis and heart ischemia reperfusion injuries [104], indicating that JAMs are important for the proper extravasation of inflammatory cells from the vasculature to the injured tissue. The same neutrophil migration process is involved in the immune response during wound healing, which suggests that JAM molecules may also play a role, especially in the inflammatory phase.

Other TJ proteins may also be involved in immune activation. In patients with multiple sclerosis, the expression levels of claudin-1 and claudin-5 are elevated in several populations of peripheral blood leukocytes, especially B and T lymphocytes and monocytes, and their expression decreases after glucocorticoid treatment [100]. Claudin-1 expression is also increased in the peripheral blood leukocytes of patients with type-1 diabetes [100]. These results suggest that elevated levels of claudins in leukocytes are associated with the activation of immune cells and could potentially be used in conjunction with other TJ molecules as new biomarkers to measure immune system activation, dysfunction, or therapeutic response in immune or auto-immune diseases. However, there does exist some debate about the relationship between neutrophils and TJ barrier activity. One interesting study by Burns shows that activated neutrophil adhesion or transmigrating across an IL-1 treated monolayer of endothelial cells does not lead to obvious disruption of the structure of TJ proteins, such as ZO-1, ZO-2, and occludin, by proteolysis, nor does it impair permeability [105], suggesting that neutrophil transmigration may not affect the barrier function of TJs in endothelial cells.

The involvement of TJ proteins in inflammation also extends to one of the most prominent locations of TJs themselves: epithelial cells, which are currently considered to be part of the innate immune system [106]. Nasal epithelial cells express all 10 known human toll-like receptors (TLRs) [107]. After TLR3 ligand poly (I:C) treatment, these cells exhibit significant down-regulation of JAM-A and pro-inflammatory cytokines interleukin-8 and tumor necrosis factor-α, which are modulated via the epidermal growth factor receptor, phosphatidyl inositol-3 kinase, p38 MAPK, and nuclear factor-κB signal transduction pathways [108]. Taken together, these results further emphasize the apparent relationships between TJ proteins, inflammation, immune response, and signal transduction pathways, which are all are critical components of the skin and its normal functions, including repair after injury. Notably, these studies also highlight the complexity of the functions of TJ proteins and the many ways in which they can be modulated. It is important to recognize that a number of other factors, including cytokines, growth factors, phosphorylation status, and miRNAs, can also regulate the expression, signaling transduction, and functions of TJ molecules. Since little information is available regarding their specific involvement in wound healing, these concepts are beyond our scope, but more information about the effects of these factors on TJ proteins is available in the referenced reviews [19,26,109,110,111,112,113,114,115].

## 9. Tight Junctions in Wound Healing

The wound healing process is comprised of several fundamental steps that must each occur properly in order for the wound to successfully heal. One such step is re-epithelialization, which is the restoration of lost epithelium by proliferation and migration of epithelial cells from the wound edge to cover the wound bed on provisional or newly formed granulation tissue. Successful re-epithelialization provides a crucial temporary barrier in the early wound healing process. Granulation tissue formation involves angiogenesis and the proliferation and differentiation of fibroblasts, which provide a solid base that allows for the development of a permanent epithelial barrier during the maturation and remodeling phases. These processes begin immediately after injury, when a combination of factors signals the activation of the tissue repair program. One such factor is the compromised integrity of epithelial cell TJs at the wound edge. This disruption of the normal epithelium induces epithelial cell proliferation and migration, which ultimately reassembles TJs to form a new, intact epithelial layer [116]. Given the significant contribution of TJs to epithelial cell barrier function, it follows readily that TJ reconstitution is an essential element of wound repair. However, as we have discussed, thorough investigation into the exact roles of TJs in wound healing has been largely neglected especially in in vivo studies. Thus far, we have discussed existing information regarding the general functions of specific TJ molecules and the data suggesting they may play a role in wound healing. In this section, we present summaries of the few in vitro and in vivo studies that have directly investigated the involvement of TJ proteins in wound healing (Table 3).

Whole tissue gene expression microarray data generated in our laboratory from mouse skin wounds at 6, 12, and 24 h and 3, 5, 7, and 10 days post-wounding shows that multiple TJ molecules, including occludin; claudins 1, 3, 4, 7, 10, 11, 12, 14, and 23; ZOs-1, 2, 3; and JAMs-A, B, and C; undergo significant dynamic changes in expression over the course of wound healing. By comparison, oral wounds demonstrate an overlapping but distinct pattern of expression that includes occludin; claudins 1, 2, 4, 5, 11, 12, 13, 15, 18, 19, and 23; ZO-2; and JAMs-A, B, and C. In addition, the levels of gene expression of occludin, JAM-A, and claudins 1, 3, 10, 15, and 23 in normal skin are significantly higher than in a normal tongue. Furthermore, gene expression of certain TJ molecules differs in human skin and oral keratinocytes in response to injury or lipopolysaccharide treatment, which suggests that there is differential expression of TJs in both normal and wounded skin and oral mucosa [117]. These data further highlight the significant relationship between differential wound healing and differential TJ protein expression and function in the skin as compared to the oral mucosa.

An experimental epidermal wound model to investigate the production and distribution of TJs revealed expression of Claudin-1, occludin, and ZO-1 in the leading edge of the ingrown epithelial cells prior to the reconstruction of the stratum corneum [66]. One study shows that peptidoglycan, which is a TLR2 agonist, enhances the barrier function of human skin keratinocytes, as indicated by an increase in TEER and significant increases in the expression levels of claudin-1, claudin-23, occludin, and ZO-1. In addition, peptidoglycan treatment also promotes the skin barrier recovery in epidermal wounds inflicted by tape stripping [118]. A scratch wound model using a cultured monolayer of MDCK cells also showed that occludin localized at the leading edge of migrating epithelial cells. Knocking down occludin impaired cell migration and polarization of epithelial cells by disrupting the accumulation of aPKC-Par3 and protein associated to tight junctions (PATJ) at the leading edge, resulting in a disorganized microtubule network. These results indicate that aPKC-Par3 and PATJ play important roles in the polarization of epithelial cells by recruiting junction proteins [119]. Together, these studies draw attention to the variety of TJ proteins that are present in the leading edge during wound closure and further emphasize the potential consequences of TJ protein dysfunction in the healing wound.

In a human skin suction blister model, water evaporation from the wound area is significantly increased, indicating decreased epidermal barrier function [120]. Occludin and ZO-1 are present in the granular cell layer of the epidermis close to the blister. In the hyper-proliferative area surrounding the border of the blister, the expression of ZO-1 extends into a few layers of spinous cells. However, occludin is limited to the upper epidermis. Both occludin and ZO-1 proteins are only present in keratinocytes at the leading edge of migrating cells. As the blister wound heals, water evaporation from the wound area decreases, while the expression of these TJ molecules increases. This highlights a direct relationship between wound healing and the presence of TJ proteins. Interestingly, the same study reports that tape stripping of the corneal layer of human skin has no effect on the expression of ZO-1 and occludin [120], which suggests that TJ molecules may respond differently to different types of injury.

In an ex vivo acute human skin wound model, claudin-1 was identified in all layers of the epidermis, while occludin was restricted to the stratum granulosum, the keratinocytes at the regenerating wound margin, and unwounded areas [121]. However, in the tissue surrounding the wound area of chronic leg ulcers, both claudin-1 and occludin are absent in a substantial portion of the regenerating epidermis at the wound edge [121], which suggests that the absence of certain TJ molecules may contribute to poor healing in chronic wounds. When cultured in low-calcium conditions, keratinocytes mimic the behavior of the cells at the leading edge of the wound margin, which proliferate but do not readily differentiate and, therefore, do not form mature TJs, even though the TJ component molecules are present. In these low calcium conditions, siRNA knockdown of claudin-1 markedly attenuates phosphorylation of protein kinases AKT and ERK1/2 and decreases the proliferation and migration of human primary skin keratinocytes, resulting in impaired wound healing [121]. Since these undifferentiated, proliferative, migrating keratinocytes at the wound edge are important for wound closure, these studies suggest that claudin-1 has important functions in wound healing beyond its role in the barrier function of mature TJ complexes [121]. The authors also studied wound closure in the same claudin-1 knockdown model under high-calcium conditions, which allow cellular differentiation and promote the assembly of mature TJs. The results show that wound closure is slower in high-calcium conditions than in low-calcium conditions [121]. Taken together, these results demonstrate that claudin-1 may be required for proper wound closure in both proliferating and differentiating keratinocytes. In both populations, the absence of claudin-1 impairs wound closure, which indicates that it may play crucial roles both as an independent protein and within the TJ complex.

Mesenchymal stem cells (MSCs) are non-hematopoietic, multipotent, stromal cells that can secrete paracrine factors and differentiate into several cell types in the skin, including keratinocytes, endothelial cells, pericytes, and fibroblasts [122]. Numerous animal studies and human clinical trials demonstrate that MSCs improve skin wound healing due to their anti-inflammatory and anti-microbial activity, as well as the production of a variety of growth factors and pro-angiogenic factors [123]. In a mouse model, overexpression of JAM-A in MSCs promotes the migration of MSCs to hair follicles and enhances hair growth and formation [124]. JAM-A overexpression also accelerates skin wound closure, increases the proliferation and migration of epithelial cells, and promotes angiogenesis. In addition, JAM-A overexpression in MSCs enhances their proliferative activity and secretory function and activates the Tiam1/Rac1 signaling pathway, which increases homing/chemotaxis and migration [125]. Taken together, these data highlight JAMs as a potential contributor to the positive effects of MSCs on wound healing. In addition to the application of MSCs, negative-pressure wound therapy is another common treatment for chronic wounds. In an in vitro study using HACAT and MDCK cells, negative-pressure enhanced wound closure, possibly by contributing to the disassembly of ZO-1 and enhancing epithelial migration [126]. This result suggests that decreased ZO-1 may promote faster wound closure, highlighting ZO-1 as another potential therapeutic target in chronic non-healing wounds.

Other studies have investigated TJ expression in non-skin tissues during wound repair. Corneal epithelial wounds in type 2 diabetic rats heal more slowly and exhibit decreased occludin expression as compared to wild type rats, indicating that abnormal occludin expression may contribute to delayed epithelial wound healing in the diabetic cornea [127]. Furthermore, an electron microscopy study of junctional molecules in guinea pig oral hard palate tissue samples taken 18, 48, 96, and 120 h after wounding shows that desmosomes and gap junctions are present in the wound epithelium throughout the observation period. In the same tissue, however, TJs are absent or fragmentary at best [128]. In a mouse ex vivo superficial or full-thickness urothelium wound model, the expression of occludin is disrupted in the superficial cells immediately after wounding. However, a punctate and patchy pattern of occludin expression appears in the cell–cell junctions between the original superficial cells and new superficial cells 1 h after injury. As healing progresses, the expression pattern of occludin gradually forms a continuous line between the original and new superficial cells 24 h after injury. The same study also shows that occludin-containing-TJs develop before the newly generated superficial epithelial cells are terminally differentiated, indicating that the original superficial epithelial cells play an important role in urothelial wound repair by forming shared occludin-containing TJs with the de-novo differentiated epithelial cells [129]. This distinctive pattern of TJ expression highlights their importance during wound healing and further underscores the need for a deeper understanding of their specific functions.

## 10. Take-Home Messages

- Bi-cellular tight junctions are functional intercellular junctions between two adjacent cells and are formed by various transmembrane and intracellular scaffold proteins. They are critical structures and have two especially important roles: the fence function, which maintains apical polarity, and the barrier function, which controls the paracellular passage of ions and molecules. The most extensively studied bi-cellular TJ proteins are occludin, claudins, JAMs, and ZOs.
- TJs are primarily present in epithelial cells and endothelial cells. However, some immune cells, such as lymphocytes and monocytes, also express TJs. For some TJ component proteins, evidence exists that they may have important functions in the immune response.
- Limited information is available regarding the specific functions of TJs and their component proteins in wound healing. Some studies show that claudin-1, occludin, and ZO-1 are present in migrating keratinocytes at the wound edge and that claudin-1 and occludin are absent in the epithelial cells of chronic skin wounds. Additionally, overexpression of JAM-A in MSCs enhances their homing to skin wounds and improves wound closure, angiogenesis, and the proliferation and migration of epithelial cells during wound healing.
- A better understanding of the functions of TJs during wound healing, including their involvement in the barrier function; re-epithelialization; angiogenesis; cell proliferation; cell migration; the inflammatory response; and the interactions between epithelial cells, endothelial cells, and immune cells may elucidate novel targets for improving tissue repair.

## 11. Conclusions

Bi-cellular tight junctions are a type of junctional complex primarily located between individual epithelial and endothelial cells. They are important functional protein complexes involved in fence and barrier function, proliferation, migration, differentiation, and signal transduction in epithelial cells and endothelial cells, as well as in the regulation of the immune response. Limited information is available regarding the specific functions of TJs in wound healing, but some studies suggest that TJs are expressed by migrating epithelial cells at the wound edge and may be absent in chronic wounds [121]. Because wounds are open during the early stages of wound healing, molecules or microorganisms can move freely through the compromised tissue surface. This suggests that TJ barrier function may not be critical prior to the completion of re-epithelialization. However, the various TJ proteins expressed by epithelial cells at the wound edge or in the wound area may contribute to the regulation of cell proliferation, differentiation, and migration during this process, independently or by interacting with neighboring cells, such as resident immune cells and infiltrating inflammatory cells. Once the healing tissue enters the remodeling phase, the wound itself closes and the barrier function of TJs in the epithelial cells may then become more essential for proper healing and remodeling.

Our understanding of the involvement and function of TJs in wound healing is still in its early stages, and much remains to be explored. How do TJs regulate the proliferation, migration, and differentiation of epithelial cells before wound closure? How do the functions of epithelial cell TJs change during the remodeling phase? What are the signals that lead to those changes? How do the functions of TJs differ throughout the different stages of wound healing? How does the expression and function of TJs affect the inflammatory response in healing and non-healing wounds? Are TJs involved in scar formation and remodeling? How does the interaction between epithelial and endothelial cell TJs and immune cells modulate cell function? As we better understand the roles TJs play in wound healing, we can begin to decipher the mechanisms behind non-healing wounds and scar formation and address new therapeutic targets for optimal healing. The answers to these key questions will not only further our knowledge of cell-cell interactions but also unlock the potential for advancement in wound care.

## Figures and Tables

**Table 1:** Distribution of bi-cellular tight junction proteins in the human and mouse skin epidermis.

TJ Protein	Stratum Basale	Stratum Spinosum	Stratum Granulosum	Stratum Corneum	References
**Human**					
Claudin-1	+	+	+	+	[66,73,78,79,80]
Claudin-2	−	−	+	−	[81]
Claudin-3	−	−	(+)	−	[82]
Claudin-4	−	+	+		[68,75,83]
Claudin-5	−	−	(+)	−	[84]
Claudin-7	(+)	+	+	−	[2]
Claudin-17	−	−	+	−	[68]
Occludin	−	−	+	+	[79,85,86,87]
JAM-A	+	+	+	−	[2]
ZO-1	−	−/+	+	−	[80,85]
Cingulin	−	−	+	−	[2]
**Mouse**					
Claudin-1	+	+	+	−	[69,73]
Claudin-4	−	−/+	+	−	[69,88]
Claudin-6	−	−/+	+	−	[89]
Claudin-10	−	−	−	+	[89]
Claudin-12	+	+	+	−	[89]
Claudin-18	−	−/+	+	−	[67]
Occludin	−	−	+	−	[67]
JAM-A	+	+	+	−	[90]
ZO-1	−	−/+	+	−	[67,85]
ZO-2	−	−/+	+	−	[67]

+: Positive, (+): Weakly positive, −: Negative; −/+ Found in upper stratum spinosum but not in the lower stratum spinosum. Adopted from reference [3]. JAM: junctional adhesion molecule; ZO: zonula occludens.

**Table 2:** Distribution of bi-cellular tight junction proteins in human oral mucosa.

TJ Protein	Stratum Basale	Stratum Spinosum	Stratum Granulosum	Stratum Corneum	References
Claudin-4	+	+			[93]
Claudin-15	+	+			[93]
Occludin	+	+	+		[93]
ZO-1	+	+	+		[93,99]
ZO-2	+	+	+		[93]
Occludin	+	+	+		[93]
JAM-A	+	+	+		[93]

Note: ZO-1 was examined in both gingival and buccal tissues. All other molecules were studied in gingival tissue only.

**Table 3:** Summary of findings regarding bi-cellular tight junction molecules in various wound models.

TJ Molecules	Wound Model	Findings	References
**Skin or Skin Epithelial Cells**			
Claudin-1 Occludin ZO-1	3-D human skin keratinocyte culture	Claudin-1, occludin, and ZO-1 are present in the first of the ingrowing epithelial cells.	[66]
Occludin ZO-1	(A) Human skin suction blister (B) Tape stripping	Occludin and ZO-1 are present in keratinocytes at the leading edge of migrating cells. Water evaporation from the wound area is decreased. Tape stripping has no effect on ZO-1 and occludin expression.	[120]
Claudin-1 Claudin-2 Claudin-3 Occludin ZO-1	Human skin tape stripping	TLR2 agonist enhances expression of claudins 1 and 23, occludin, and ZO-1 in keratinocytes, increases skin barrier recovery, and decreases paracellular flux.	[118]
JAM-A	Mouse, 5 mm punch wound	JAM-A overexpression in MSCs enhances homing of MSCs to skin wounds, wound closure, proliferation, migration of epithelial cells, and angiogenesis. JAM-A over expression in MSCs also increases their proliferative activity and secretory function.	[125]
Claudin-1 Occludin	(A) Human chronic leg ulcer (B) Human skin keratinocyte scratch wound	Claudin-1 and occludin are largely absent in the regenerating epidermis at the wound edge. Knockdown of claudin-1 attenuates proliferation and migration of keratinocytes.	[121]
**Other Tissues or Non-Skin Epithelial Cells**			
TJs	Guinea pig oral hard palate wound	When visualized by electron microscopy, intact desmosomes and gap junctions are observed in the wound epithelium. However, TJs are absent or fragmentary at best.	[128]
Occludin	Human ex vivo superficial or full-thickness urothelium wounds	A continuous line of occludin expression is observed between the original and new superficial epithelial cells 24 h after injury. Occludin is expressed before the newly generated superficial epithelial cells are terminally differentiated.	[129]
Occludin	MDCK Scratch wound	Occludin localizes to the leading edge of migrating epithelial cells. Occludin knockdown impairs epithelial cell migration and polarization.	[119]
ZO-1	MDCK, Scratch wound, Negative-pressure treatment	Negative-pressure enhances wound closure possibly by disassembling ZO-1 to enhance epithelial migration.	[126]
Occludin	Diabetic rat 4 mm circular corneal epithelial wound	Corneal epithelial wounds demonstrate decreased occludin expression in diabetic rats.	[127]

## Abbreviations

CAR	coxsackie and adenovirus receptor
DETC	dendritic epidermal γδ T cells
JAM	junctional adhesion molecule
MAGUK	membrane-associated guanylate kinase
MAPK	mitogen-activated protein kinase
MDCK	madin-Darby canine kidney epithelial cell line
MSCs	mesenchymal stem cells
PDZ	post-synaptic density 95/Drosophila discs large/zona-occludens
PK	protein kinase
TEER	transepithelial/endothelial electrical resistance
TJ	tight junction
TLR	toll-like receptor
ZO	zonula occludens
